# Considerations for the Analysis of Bacterial Membrane Vesicles: Methods of Vesicle Production and Quantification Can Influence Biological and Experimental Outcomes

**DOI:** 10.1128/Spectrum.01273-21

**Published:** 2021-12-22

**Authors:** Natalie J. Bitto, Lauren Zavan, Ella L. Johnston, Timothy P. Stinear, Andrew F. Hill, Maria Kaparakis-Liaskos

**Affiliations:** a Department of Physiology, Anatomy and Microbiology, La Trobe University, Melbourne, Victoria, Australia; b Research Centre for Extracellular Vesicles, School of Molecular Sciences, La Trobe University, Melbourne, Victoria, Australia; c Department of Microbiology and Immunology, Doherty Institute, University of Melbourne, Parkville, Victoria, Australia; d Department of Biochemistry and Genetics, La Trobe Institute for Molecular Science, La Trobe University, Melbourne, Victoria, Australia; University of Florida

**Keywords:** bacterial membrane vesicles (BMVs), DNA, membrane vesicles (MVs), NTA, outer membrane vesicles (OMVs), RNA, TLRs, protein, quantification

## Abstract

Bacterial membrane vesicles (BMVs) are produced by all bacteria and facilitate a range of functions in host-microbe interactions and pathogenesis. Quantification of BMVs is a critical first step in the analysis of their biological and immunological functions. Historically, BMVs have been quantified by protein assay, which remains the preferred method of BMV quantification. However, recent studies have shown that BMV protein content can vary significantly between bacterial strains, growth conditions, and stages of bacterial growth, suggesting that protein concentration may not correlate directly with BMV quantity. Here, we show that the method used to quantify BMVs can alter experimental outcomes. We compared the enumeration of BMVs using different protein assays and nanoparticle tracking analysis (NTA). We show that different protein assays vary significantly in their quantification of BMVs and that their sensitivity varies when quantifying BMVs produced by different species. Moreover, stimulation of epithelial cells with an equivalent amount of BMV protein quantified using different protein assays resulted in significant differences in interleukin 8 (IL-8) responses. Quantification of Helicobacter pylori, Pseudomonas aeruginosa, and Staphylococcus aureus BMVs by NTA and normalization of BMV cargo to particle number revealed that BMV protein, DNA, and RNA contents were variable between strains and species and throughout bacterial growth. Differences in BMV-mediated activation of Toll-like receptors, NF-κB, and IL-8 responses were observed when stimulations were performed with equivalent BMV particle number but not equivalent protein amount. These findings reveal that the method of BMV quantification can significantly affect experimental outcomes, thereby potentially altering the observed biological functions of BMVs.

**IMPORTANCE** Recent years have seen a surge in interest in the roles of BMVs in host-microbe interactions and interbacterial communication. As a result of such rapid growth in the field, there is a lack of uniformity in BMV enumeration. Here, we reveal that the method used to enumerate BMVs can significantly alter experimental outcomes. Specifically, standardization of BMVs by protein amount reduced the ability to distinguish strain differences in the immunological functions of BMVs. In contrast, species-, strain-, and growth stage-dependent differences in BMV cargo content were evident when BMVs were enumerated by particle number, and this was reflected in differences in their ability to induce immune responses. These findings indicate that parameters critical to BMV function, including bacterial species, strain, growth conditions, and sample purity, should form the basis of standard reporting in BMV studies. This will ultimately bring uniformity to the field to advance our understanding of BMV functions.

## INTRODUCTION

The secretion of bacterial components via membrane-bound vesicles, collectively termed bacterial membrane vesicles (BMVs), is a phenomenon observed ubiquitously across all bacterial species (1). BMVs are produced by Gram-negative and Gram-positive bacteria via different mechanisms (1). Gram-negative bacteria produce vesicles derived from the bacterial outer membrane, thereby termed outer membrane vesicles (OMVs) (2). In contrast, Gram-positive bacteria lack an outer membrane, and thus vesicles released from their cytoplasmic membrane are simply termed membrane vesicles (MVs) (3). Both OMVs and MVs contain a range of microbe-associated molecular patterns (MAMPs), including proteins (3, 4), peptidoglycan (5–7), RNA (7–9), DNA (7, 10, 11), lipopolysaccharide (12), and lipoproteins (13). BMVs may deliver their functional cargo to target prokaryotic or eukaryotic cells to mediate interbacterial communication or host-pathogen interactions, respectively (14). For instance, BMVs can transfer DNA to bacteria to facilitate horizontal gene transfer (15) or deliver nucleic acids into eukaryotic epithelial cells to mediate an inflammatory response (5, 7, 11, 16, 17). Similarly, BMVs containing toxins may be used to outcompete neighboring bacterial species (18, 19) or to disrupt epithelial cell barriers to facilitate host colonization, drive inflammation, and mediate pathogenesis (20–23). The multifaceted functions of BMVs have been the focus of growing attention over recent years, with increasing interest in the value of BMVs in novel therapeutic applications (24, 25).

Quantification of BMVs is a crucial step in the study of their composition and biological functions and forms the basis of all subsequent assays (26). Accurate quantification of BMVs is particularly critical for functional characterizations of BMVs, such as the examination of their immunogenic functions, roles in interbacterial communication, and development as novel BMV-based therapeutics. Historically, BMVs have been quantified based on their protein content (27), and this continues to be the preferred method of BMV quantification to date and is used in a range of studies examining the immune modulating functions of BMVs (14, 20, 24–26). Furthermore, a range of protein assays are used to quantify BMVs, including Bradford (28, 29), bicinchoninic acid (BCA) (12, 30), Lowry (31), or Qubit protein assays (32, 33), with no single protein assay used as the standard method to quantify BMVs in the field. However, variables in BMV isolation (34), bacterial strains (35), culture conditions (36), growth stage (4), and BMV size (29) can all significantly alter BMV protein content, suggesting that BMV protein concentration may not directly correlate with BMV quantity.

Nanoparticle detection tools, such as NanoSight nanoparticle tracking analysis (NTA), are routinely used in the eukaryotic extracellular vesicle field to determine the concentration and size distribution of vesicles (37) and are based on principles of light scattering and Brownian motion (38). NTA offers several advantages to protein assays as a means of BMV quantification, as it requires a very small amount of sample, provides a size distribution of particles, and most importantly, quantifies BMVs irrespective of their cargo content (39). Direct quantification of BMVs, which is not based on BMV content, is particularly important in studies where BMVs obtained from different culture conditions, bacterial species, or strains are compared, as these parameters are known to affect BMV protein content. Furthermore, basing functional assays on particle number can facilitate biological comparisons of BMV functions between studies, enabling a wider understanding of BMV functions and bringing more clarity to the field. Although recent studies have used NTA as a means of quantifying BMV production and their size distribution (13, 29, 40, 41), only a limited number of studies have used particle number as a unit of measurement of BMVs in functional assays (7, 17, 42).

To date, there has not been a detailed comparison of the biological outcomes of experiments performed using BMVs that have been quantified using the traditional method of protein assay compared to those quantified using nanoparticle counting. Furthermore, it remains unknown whether experimental outcomes may be influenced by the type of protein assay used to quantify BMVs. We show that there are differences in the quantification of BMVs using various protein assays. Furthermore, we reveal significant differences in the biological effects mediated by BMVs when performing analyses based on their quantification by particle number compared to protein concentration, in addition to when using different protein assays. We examined strain-, species-, and growth stage-dependent differences in the amount, composition, and immunogenic properties of BMVs produced by the Gram-negative and Gram-positive species Helicobacter pylori, Pseudomonas aeruginosa, and Staphylococcus aureus. We demonstrate that performing immunological assays using an equivalent amount of BMVs from each organism quantified based on their protein concentration can conceal variations in the amount of immunogenic cargo carried by BMVs, and this significantly affects analyses of their immunostimulatory properties. In contrast, performing the same assays using an equivalent amount of BMVs quantified by particle number revealed significant differences in their ability to be detected by pattern recognition receptors (PRRs), activate nuclear factor kappa-B (NF-κB), and induce a proinflammatory response. These differences in the immunogenic properties of BMVs corresponded with the amount of immunogenic protein, DNA, and RNA cargo they contained, highlighting the importance of considering variations in the quantity of immunogenic cargo carried by BMVs. These findings reveal that strain-, species-, and growth stage-dependent differences in BMV cargo amount influence the immunogenic functions of BMVs, and these differences are not as well differentiated when functional assays are performed based on BMV protein concentration. In contrast, since nanoparticle counting is not dependent on BMV cargo content, assays performed based on particle number have the potential to reveal subtle differences in the biological or immunogenic functions of BMVs. These findings highlight the limitations of performing biological assays based on BMV protein concentration, in particular when comparing the functions of BMVs that may differ in their cargo content. Therefore, we propose a standardized, integrative approach to BMV quantification, whereby several parameters that influence BMV function are also reported, including bacterial stage of growth, growth conditions, sample purity, particle number, BMV size, and cargo content. The standardization of BMV quantification will facilitate biological comparisons of the functional differences between BMVs across different bacterial species, strains, growth conditions, and biological studies and will ultimately bring consistency and comparability to the field of BMV research.

## RESULTS

### Variations in protein assays affect the quantification of BMVs and the outcomes of downstream functional assays.

**FIG 1:**
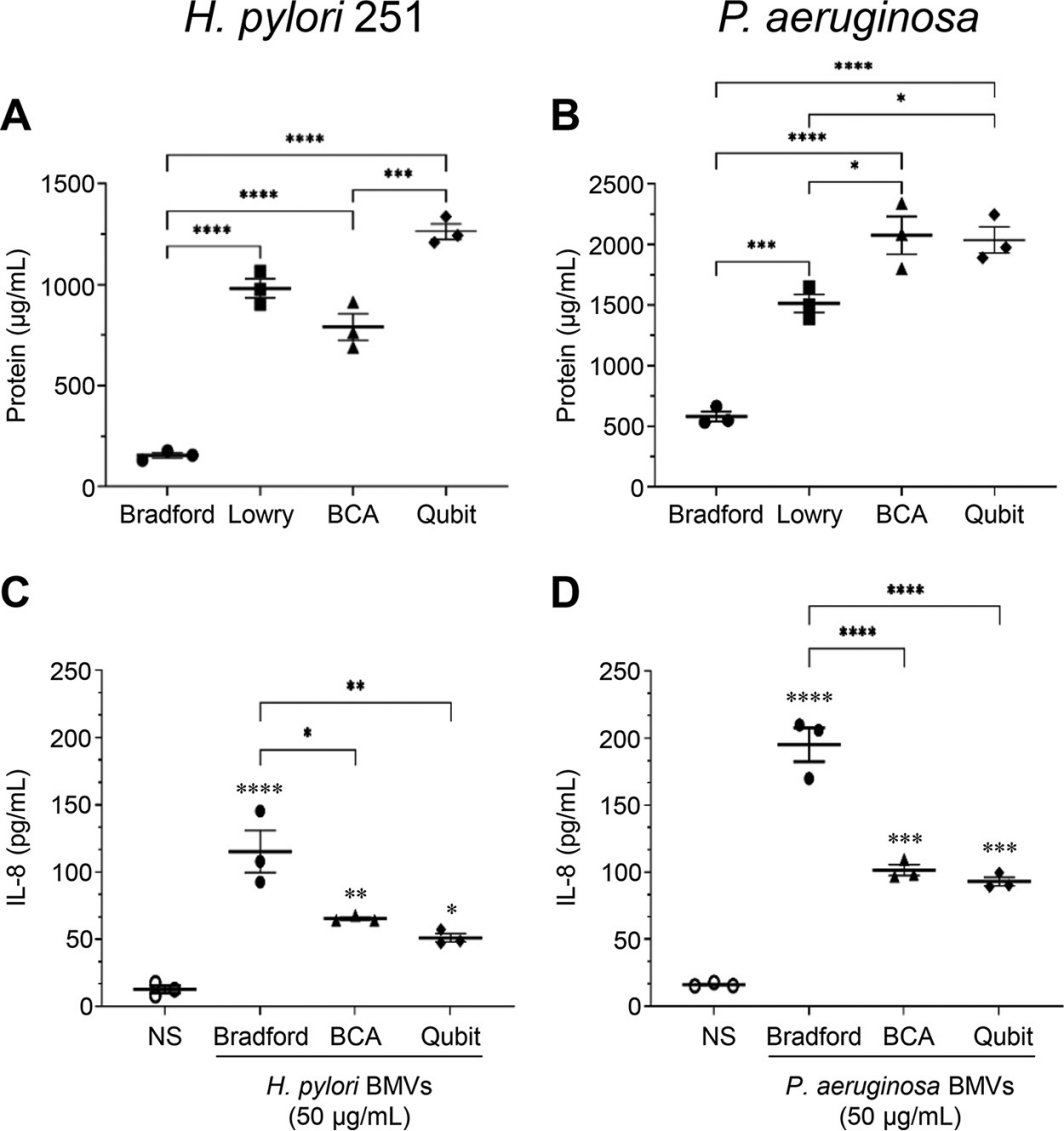
Variations in protein assay sensitivity significantly affect the quantification of BMVs and the outcome of downstream functional assays. Quantification of BMVs from (A) H. pylori 251 and (B) P. aeruginosa, using Bradford (circles), Lowry (squares), BCA (triangles), and Qubit (diamonds) protein assays. AGS cells were stimulated with 50 μg/mL of (C) H. pylori 251 BMVs or (D) P. aeruginosa BMVs that were quantified using Bradford (filled circles), BCA (triangles), or Qubit (diamonds) protein assays, and IL-8 production was measured by ELISA. Nonstimulated AGS cells served as a negative control (NS; open circles). Data are the mean ± SEM of *n* = 3 biological replicates. *, *P* < 0.05; **, *P* < 0.01; ***, *P* < 0.001; ****, *P* < 0.0001 (one-way ANOVA compared to the nonstimulated [NS] control [0 BMVs/mL; open circles] unless indicated with brackets).

The most widely used method to quantify BMVs is indirect and involves quantifying their protein content using colorimetric protein assays, such as Bradford (28, 41), bicinchoninic acid (BCA) (9), and Lowry (31) assays, or fluorometric assays, such as Qubit protein assay (32). To investigate whether there are significant differences in the enumeration of BMVs using different protein assays, we quantified H. pylori 251 BMVs and P. aeruginosa BMVs using Bradford assay, which detects proteins using Coomassie dye (43), the copper-based Lowry and BCA assays (44), and the fluorometric Qubit assay (Fig. 1A and B). We observed significant differences in the quantification of BMVs using each protein assay (Fig. 1A and B). Specifically, Bradford assay resulted in significantly lower BMV protein quantification compared to Lowry, BCA, and Qubit for both H. pylori 251 BMVs and P. aeruginosa BMVs (Fig. 1A and B). Furthermore, we also observed species-dependent differences in the quantification of BMVs using different colorimetric protein assays (Fig. 1A and B). Specifically, the BCA assay resulted in a significantly higher protein concentration of P. aeruginosa BMVs compared to the Lowry assay (Fig. 1B; *P* = 0.0208); however, there was no difference in the quantification of H. pylori BMVs by BCA compared to that by Lowry (Fig. 1A; *P* = 0.0658). This demonstrates that BMV quantification based on their protein content varies significantly due to the type of protein assay used and that there is variability within the same protein assay when quantifying BMVs produced by different bacterial species.

To determine whether these differences in BMV protein enumeration affect the outcome of downstream immunological assays, we incubated human gastric epithelial (AGS) cells with 50 μg/mL of H. pylori 251 BMVs or P. aeruginosa BMVs that were quantified using Bradford, BCA, or Qubit protein assays and detected interleukin-8 (IL-8) production using enzyme-linked immunosorbent assay (ELISA; Fig. 1C and D). We observed a significant increase in IL-8 production by AGS cells stimulated with 50 μg/mL H. pylori 251 BMVs or P. aeruginosa BMVs that were quantified by Bradford, compared to that by cells stimulated with 50 μg/mL BMVs quantified using BCA or Qubit protein assays (Fig. 1C and D). These findings show that the type of protein assay used can influence the quantification of BMVs and therefore subsequent biological analyses of their immunological functions. These findings highlight the importance of considering the method of BMV quantification when examining BMV function and suggest that a standard method of BMV quantification is needed to facilitate uniformity, experimental reproducibility, and the comparison of experimental outcomes across different BMV studies.

### The production, size distribution, and protein cargo quantity of BMVs vary between bacterial species and strains.

We have previously shown that the overall protein composition of BMVs varies between different bacterial strains (7), throughout various stages of bacterial growth (4, 45), and based on BMV size (29). It has also been shown that bacterial culture conditions (46) and methods of BMV isolation (34, 47) can alter BMV protein composition. However, it is unclear whether there is variability in the overall quantity of proteins packaged into BMVs produced by different bacterial species and strains and the biological consequences of quantifying BMVs based on their variable protein cargo amounts.

**FIG 2:**
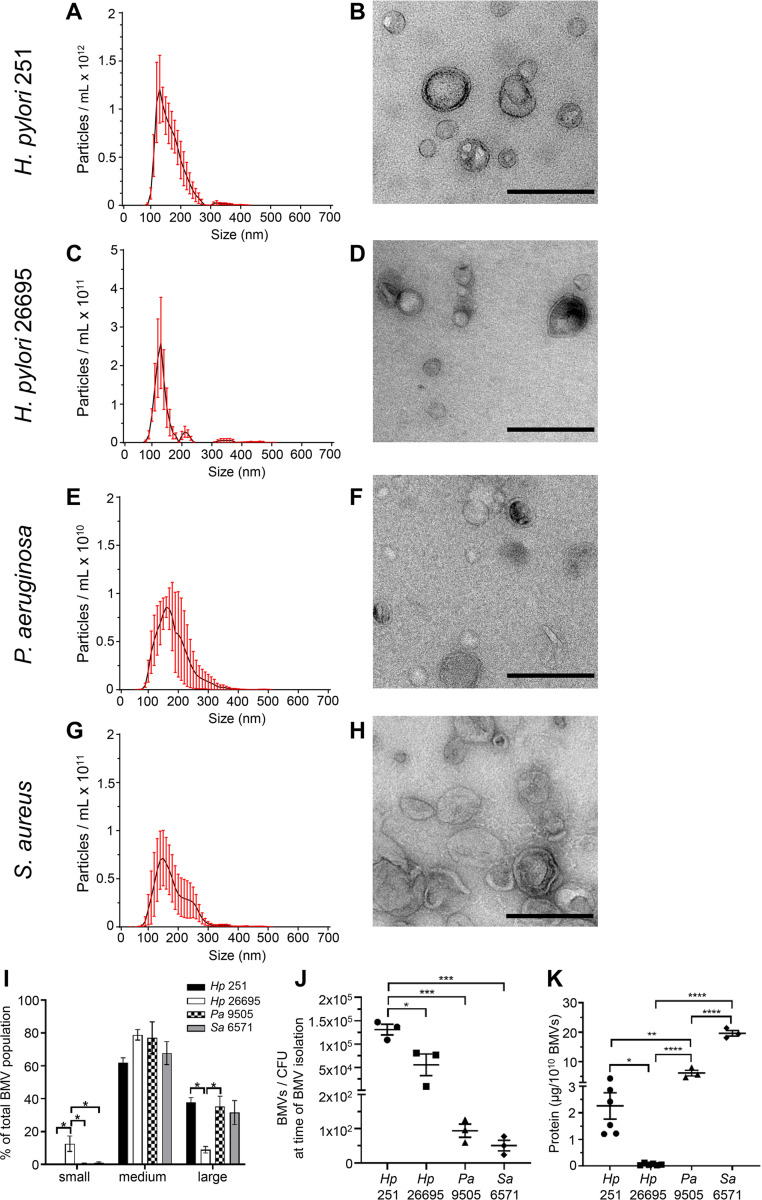
BMV quantity, size, and amount of protein cargo varies within and between bacterial species. The quantity and size distribution of BMVs produced by H. pylori 251, H. pylori 26695, P. aeruginosa PAO9505, and S. aureus 6571 were examined using NanoSight and TEM, respectively (A–H). NTA data show the mean (black line) ± SEM (red error bars) of three biological replicates. TEM images are representative of *n* = 3 biological samples. Scale bars = 200 nm. (I) Quantification by NanoSight of the size distribution of small (<100 nm), medium (100 to 200 nm), and large (>200 nm) BMVs present within heterogenous BMV samples produced by H. pylori 251 (*Hp* 251; black bars), H. pylori 26695 (*Hp* 26695; white bars), P. aeruginosa PAO9505 (*Pa* 9505; checkered bars), and S. aureus 6571 (*Sa* 6571; gray bars). Data shown is the average of three biological replicates ± SEM. *, *P* < 0.05 (one-way ANOVA with Tukey’s multiple-comparison test within each size). (J) Number of BMVs per CFU present in individual cultures for each bacterial strain at the time BMVs were isolated was determined by NanoSight NTA. Data are the mean ± SEM of *n* = 3 biological replicates. *, *P* < 0.05, ***, *P* < 0.001 (one-way ANOVA with Tukey’s multiple-comparison test). (K) Comparison of the amount of protein in BMVs produced by H. pylori 251 (*Hp* 251, circles), H. pylori 26695 (*Hp* 26695, squares), P. aeruginosa PAO9505 (*Pa* 9505, triangles), and S. aureus 6571 (*Sa* 6571, diamonds), quantified by Qubit and normalized to 1 × 10^10^ BMVs. Data show *n* ≥ 3 biological replicates with mean ± SEM. *, *P* < 0.05; **, *P* < 0.01; ****, *P* < 0.0001 (one-way ANOVA with Tukey’s multiple-comparison test).

To address this, BMVs produced by the Gram-positive species S. aureus and the Gram-negative species P. aeruginosa, H. pylori 251, and H. pylori 26695 were quantified using NanoSight NTA and visualized by transmission electron microscopy (Fig. 2A to H). Examination of the size distribution of BMVs by NanoSight revealed that all bacterial species produced BMVs of approximately 100 to 400 nm in size, with slight differences in their size distribution between strains, indicating heterogeneity in the size of BMVs they produce (Fig. 2). Specifically, H. pylori 26695 BMVs had a narrow size profile evidenced by the predominant peak ranging from 100 to 200 nm in size (Fig. 2C), compared to that of BMVs produced by the H. pylori 251 strain (Fig. 2A) and all other bacterial species examined (Fig. 2E and G). Size distribution analysis confirmed that H. pylori 26695 BMVs were significantly smaller than all BMVs examined, revealing significant differences in the size of BMVs produced by different bacteria (Fig. 2I). Quantification of the number of BMVs produced per CFU present in the bacterial cultures from which BMVs were collected showed that H. pylori 251 produced significantly more BMVs than the other species examined and that there were strain differences, as H. pylori 251 produced significantly more BMVs than H. pylori 26695 (Fig. 2J). Taken together, these findings demonstrate both strain-dependent and species-dependent differences in the amount and size of BMVs produced.

Differences in the amount and size of BMVs produced by all strains were also reflected in the quantity of their protein cargo. Quantification of BMV protein using Qubit and analysis of protein quantity per 1 × 10^10^ BMVs revealed that BMVs differed significantly in the quantity of their protein cargo within strains (Fig. 2K). Specifically, H. pylori 251 BMVs contained significantly more protein than H. pylori 26695 BMVs (Fig. 2K; *P* = 0.0239) and were also larger in size (Fig. 2I; *P* = 0.0159), correlating with our previous finding that smaller OMVs contain fewer proteins (29). There were also significant differences in the quantity of protein cargo of BMVs produced by all bacteria examined (Fig. 2K). Taken together, these findings demonstrate both strain- and species-dependent differences in the amount and size of BMVs produced and the overall quantity of their protein cargo, further highlighting the lack of correlation between protein content and BMV number.

### The quantity of BMV-associated DNA and RNA varies between bacterial species and strains.

**FIG 3:**
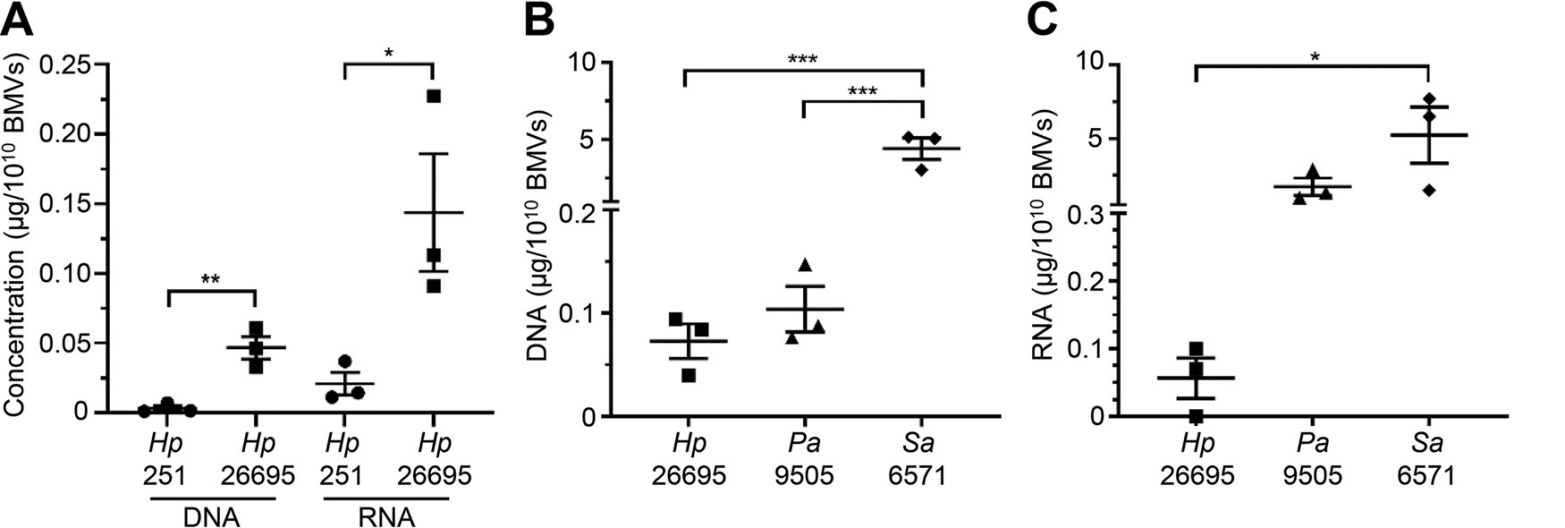
The amount of DNA and RNA in BMVs differs within and between bacterial species. (A) Interstrain comparison of the amount of DNA and RNA associated with BMVs from H. pylori 251 (*Hp* 251, circles) and H. pylori 26695 (*Hp* 26695, squares), normalized to 1 × 10^10^ BMVs. Data show *n* = 3 biological replicates with mean ± SEM. *, *P* < 0.05; **, *P* < 0.01 (unpaired *t* test). Comparison of the amount of (B) DNA and (C) RNA cargo of BMVs isolated from H. pylori 26695 (*Hp* 26695, squares), P. aeruginosa PAO9505 (*Pa* 9505, triangles), and S. aureus 6571 (*Sa* 6571, diamonds), normalized to 1 × 10^10^ BMVs. Data show *n* = 3 biological replicates with mean ± SEM. *, *P* < 0.05; ***, *P* < 0.001 (one-way ANOVA with Tukey’s multiple-comparison test).

In addition to protein, BMVs contain a range of MAMPs, including DNA, RNA, and peptidoglycan (5, 7, 11, 17). Having demonstrated the variability in the amount of protein contained by BMVs, we next sought to investigate whether other immunogenic contents of BMVs vary between bacterial strains and species. To do this, we quantified the DNA and RNA concentration of BMVs produced by S. aureus, P. aeruginosa, H. pylori 251, and H. pylori 26695 and normalized their concentration to 1 × 10^10^ BMVs. This revealed significant differences in the amount of DNA and RNA cargo of BMVs produced by different bacterial strains (Fig. 3A) and by different species (Fig. 3B and C). Specifically, we observed significant differences in the amount of DNA and RNA cargo associated with 1 × 10^10^ BMVs produced by different strains of H. pylori, as H. pylori 26695 BMVs contained significantly more DNA and RNA than H. pylori 251 BMVs (Fig. 3A; *P* = 0.0066 and *P* = 0.0462, respectively). Interspecies differences in the amount of BMV-associated DNA and RNA were also detected, as S. aureus BMVs were associated with significantly more DNA than H. pylori 26695 BMVs and P. aeruginosa BMVs (Fig. 3B; *P* = 0.006 and *P* = 0.006, respectively) and more RNA than H. pylori 26695 BMVs (Fig. 3C; *P* = 0.0431). Collectively, these findings highlight that the amount of BMV-associated cargo varies significantly within and between bacterial species and reveal the limitations of BMV quantification based on their cargo.

### The quantity of BMV-associated protein, DNA, and RNA varies throughout bacterial growth.

**FIG 4:**
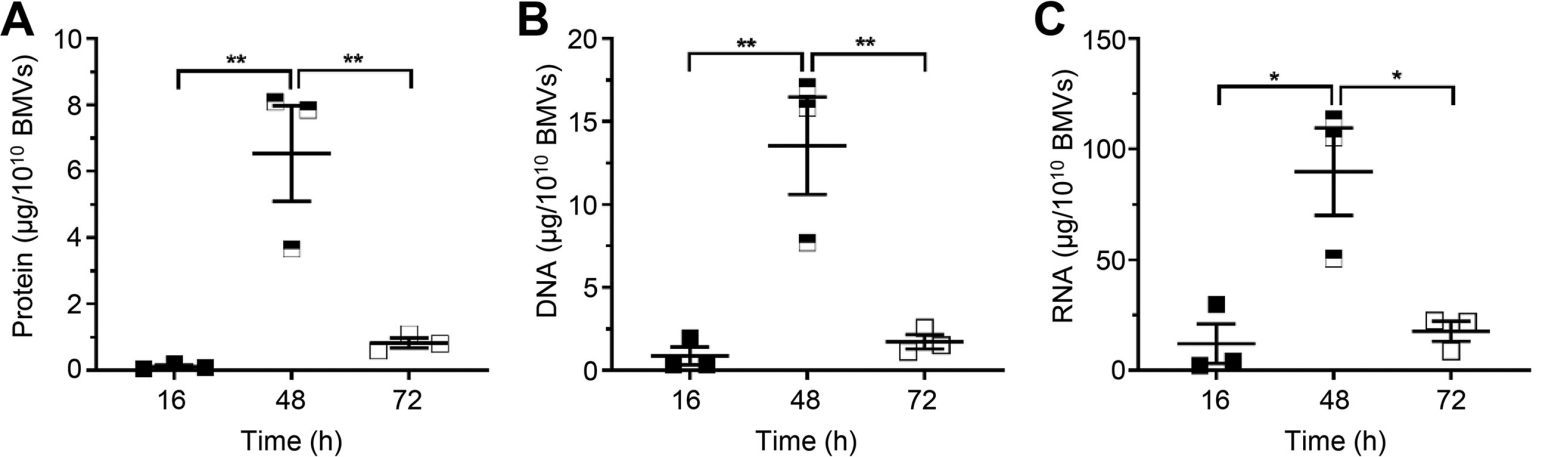
The amount of protein, DNA, and RNA associated with BMVs differs with bacterial growth stage. Comparison of the amount of (A) protein, (B) DNA, and (C) RNA (all quantified by Qubit) in H. pylori 26695 BMVs produced during early-exponential (16 h; filled squares), late-exponential (48 h; half black, half white squares) and stationary (72 h; white squares) stages of bacterial growth, normalized to 1 × 10^10^ BMVs. All data show *n* = 3 biological replicates with mean ± SEM. *, *P* < 0.05; **, *P* < 0.01 (one-way ANOVA with Tukey’s multiple-comparison test).

We have previously shown that H. pylori 26695 BMVs differ in size and protein composition based on the bacterial growth stage from which they were isolated (4). This implies that quantification of BMVs based on their protein content may not be accurate when they are isolated from different growth stages and suggests that bacterial growth stage may also regulate the quantity of MAMPs packaged within BMVs, such as DNA and RNA. To investigate whether bacterial growth stage affects the amount of protein, DNA, and RNA associated with BMVs, we isolated H. pylori 26695 BMVs produced during early-exponential (16 h), late-exponential (48 h), and stationary (72 h) phases of growth and quantified their contents (Fig. 4A to C). Quantification of the protein, DNA, and RNA associated with 1 × 10^10^ H. pylori 26695 BMVs isolated from each bacterial growth stage revealed marked differences in their contents (Fig. 4A to C), as BMVs isolated from late-exponential phase contained significantly more protein, DNA, and RNA than BMVs isolated from early-exponential and stationary growth phases (Fig. 4A to C). These findings highlight the significant variation in the amounts of protein and immunogenic cargo carried by BMVs during different stages of bacterial growth that may influence their immunostimulatory properties and their ability to activate PRRs.

### Stimulation of host cells with an equivalent number of BMVs reveals strain differences in their ability to activate NF-κB and induce the production of interleukin-8.

To date, the majority of studies examining the immunostimulatory properties of BMVs perform these analyses using a fixed amount of BMVs that have been quantified using protein assays (20, 25). This is in contrast to the eukaryotic extracellular vesicle (EV) field, which widely uses a range of techniques consisting of nanoparticle counting in conjunction with transmission electron microscopy (TEM) and the detection of EV-specific proteins by immunoblotting to examine and quantify the number of EVs (38). Having demonstrated the variability that exists in the quantity of protein, DNA, and RNA cargo of BMVs produced by different bacteria (Fig. 3) and during various stages of bacterial growth (Fig. 4), we sought to determine whether methods of BMV quantification can bias experimental outcomes when comparing the immunostimulatory properties of BMVs produced by different strains. To do this, we examined the ability of H. pylori 251 BMVs and H. pylori 26695 BMVs to activate NF-κB and induce interleukin-8 (IL-8) production by host epithelial cells that were stimulated with either an equivalent number of BMVs quantified by NTA or an equivalent amount of BMVs quantified using Bradford protein assay.

**FIG 5:**
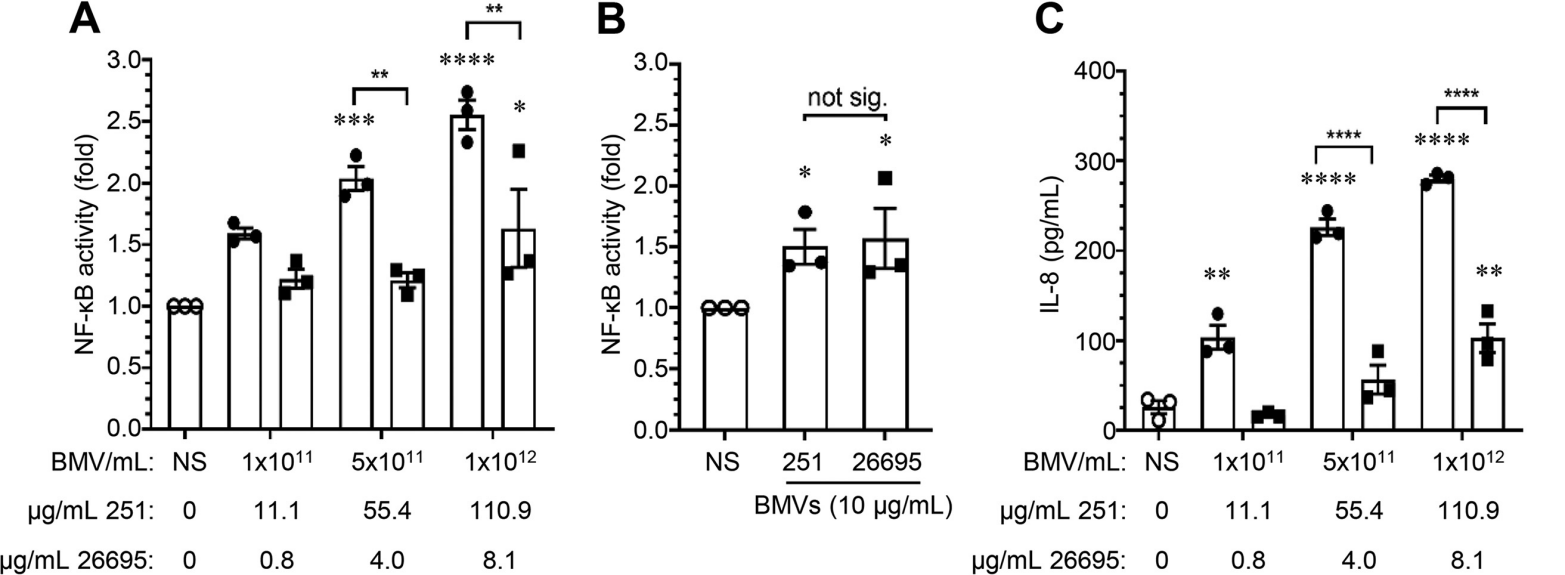
Stimulating host cells with an equivalent number of BMVs quantified by NTA reveals strain-dependent differences in their ability to activate NF-κB and induce IL-8 production. (A) HEK293 cells transiently expressing an NF-κB reporter were stimulated with equivalent amounts of early-exponential H. pylori 26695 BMVs (filled squares) or H. pylori 251 BMVs (filled circles) quantified using NanoSight NTA (BMVs/mL). The corresponding protein concentration (μg/mL) per number of BMVs was determined using Bradford assay and is also indicated. (B) HEK293 cells transiently expressing an NF-κB reporter were stimulated with 10 μg/mL of H. pylori 26695 BMVs (filled squares) or H. pylori 251 BMVs (filled circles) that were quantified using Bradford protein assay. (C) AGS cells were stimulated with an increasing number of BMVs quantified by NanoSight NTA (BMVs/mL) from H. pylori 251 (filled circles) or H. pylori 26695 (filled squares), and IL-8 production was quantified by ELISA. The corresponding protein concentration (μg/mL) determined using Bradford assay is also indicated. All data show *n* = 3 biological replicates with mean ± SEM. not sig., not significant; *, *P* < 0.05; **, *P* < 0.01; ***, *P* < 0.001; ****, *P* < 0.0001 (one-way ANOVA compared to the nonstimulated [NS] control [0 BMVs/mL; open circles] unless indicated with brackets).

First, we stimulated human embryonic kidney (HEK293) cells transiently transfected with an NF-κB luciferase construct with an increasing number of H. pylori 251 BMVs or H. pylori 26695 BMVs that were quantified using NTA (Fig. 5A) or protein assay (Fig. 5B). Significant strain differences in the ability of H. pylori 251 BMVs and H. pylori 26695 BMVs to activate NF-κB were evident when HEK293 cells were stimulated with an equivalent number of BMVs quantified by NTA (Fig. 5A) but not when they were stimulated with an equivalent amount of BMVs quantified based on their protein concentration (Fig. 5B). Specifically, H. pylori 251 BMVs quantified by NTA induced a significant level of NF-κB activation, whereas H. pylori 26695 BMVs were less effective at activating NF-κB, indicating that 251 BMVs were more immunostimulatory on a per-vesicle basis (Fig. 5A). In contrast, stimulation of HEK293 cells with an equivalent amount of H. pylori 26695 BMVs or 251 BMVs quantified based on their protein concentration revealed no difference in the level of NF-κB activation induced by BMVs produced by both H. pylori strains, therefore masking strain-dependent differences (Fig. 5B). These findings suggest that when making functional comparisons between BMVs produced by different strains and species, normalizing BMVs by protein concentration can prevent the identification of immunostimulatory and functional differences between BMVs that may be attributed to variability in their protein composition.

We next investigated whether stimulating epithelial cells with an equivalent number of BMVs revealed differences in the IL-8 response they induced. Human AGS cells were stimulated with an increasing number of H. pylori 251 BMVs or H. pylori 26695 BMVs, and IL-8 production was measured by ELISA (Fig. 5C). Consistent with our findings investigating NF-κB activation (Fig. 5A), H. pylori 251 BMVs induced significant levels of IL-8 production by AGS cells, irrespective of the number of BMV used to stimulate host cells, compared to H. pylori 26695 BMVs, which induced an IL-8 response only at the highest number of BMVs tested (Fig. 5C). Collectively, these findings highlight that standardizing the number of BMVs added to assays by particle number can allow detection of subtle differences in the functions of BMVs produced by different strains and species, while the analysis of BMVs based on equivalent amounts of BMV protein can reduce the ability to detect differences in their biological functions.

### Stimulation of host cells with an equivalent number of BMVs reveals strain differences in their ability to activate TLRs.

**FIG 6:**
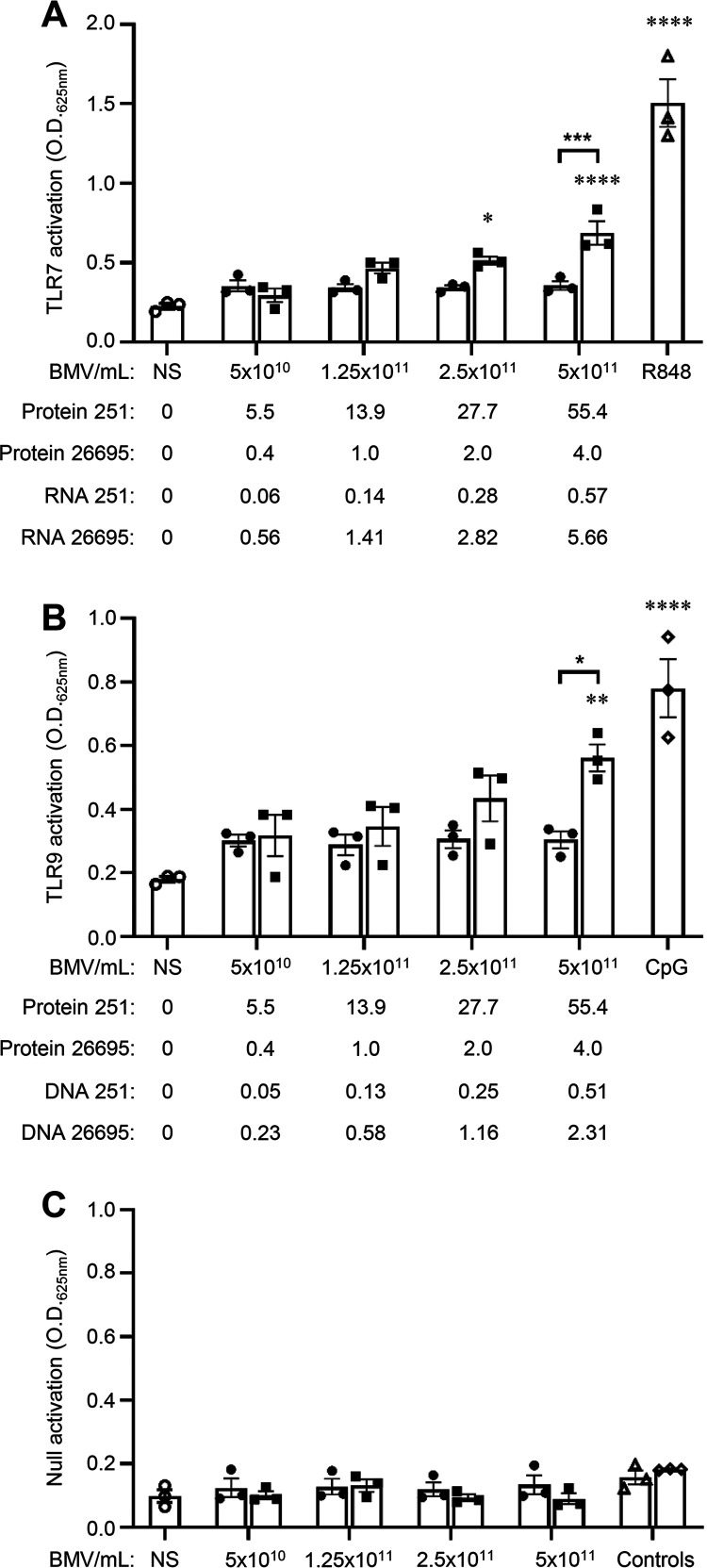
Stimulation of host cells with an equivalent number of BMVs reveals strain differences in their ability to activate TLR7 and TLR9 responses. HEK-Blue (A) TLR7, (B) TLR9, and (C) Null cells were stimulated with equivalent amounts of early-exponential H. pylori 251 BMVs (filled circles) or H. pylori 26695 BMVs (filled squares) quantified by NTA (BMVs/mL). The corresponding concentration of protein (quantified by Bradford assay), RNA, and DNA (quantified by Qubit) associated with BMVs from each strain is also indicated (μg/mL). Positive controls include 1 pg/mL R848 for TLR7 (open triangles) or 5 nm CpG-ODN for TLR9 (open diamonds), while nonstimulated (NS) cells served as a negative control (0 BMVs/mL; open circles). Data show *n* = 3 biological replicates with mean ± SEM. *, *P* < 0.05; **, *P* < 0.01; ***, *P* < 0.001; ****, *P* < 0.0001 (one-way ANOVA compared to the nonstimulated [NS] control [0 BMVs/mL; open circles] unless indicated with brackets).

Having identified that BMVs produced by different strains vary in their amount of protein, DNA, and RNA cargo, we next sought to determine whether strain differences in BMV-associated MAMP cargo altered their ability to activate Toll-like receptors (TLRs) and whether the method of BMV quantification could bias experimental outcomes. To do this, we examined whether differences in the amount of DNA and RNA carried by BMVs produced by different H. pylori strains resulted in altered TLR-mediated responses. We stimulated HEK-Blue reporter cell lines stably expressing either human TLR7 or TLR9, which recognize bacterial RNA and DNA, respectively (48, 49), or the control HEK-Blue Null cell line, with equivalent numbers of H. pylori 251 BMVs or H. pylori 26695 BMVs quantified by NTA (Fig. 6). We also quantified the corresponding amount of DNA, RNA, and protein associated with BMVs, identifying differences in their amounts associated with BMVs produced by each strain (Fig. 6). We observed significant activation of TLR7 (*P* < 0.0001) and TLR9 (*P* = 0.0038) by H. pylori 26695 BMVs but not H. pylori 251 BMVs in a dose-dependent response (Fig. 6A and B). Analysis of the corresponding DNA and RNA concentrations associated with an equivalent number of H. pylori 26695 BMVs and 251 BMVs revealed that H. pylori 26695 BMVs delivered approximately 10 times more RNA and 4 times more DNA to host cells than 251 BMVs, which is consistent with the ability of 26695 BMVs to significantly activate TLR7 and TLR9 (Fig. 6A and B). No response was detected in the control HEK-Blue Null cells stimulated with BMVs (Fig. 6C), while protein concentration had no correlation with TLR7 and TLR9 activation, suggesting that the response observed is TLR7- and TLR9-specific. Collectively, these findings demonstrate that performing biological assays based on BMV particle number can reveal differences in the ability of BMV-associated MAMPs to activate innate immune receptors and induce the production of a proinflammatory response that would not be evident if BMVs were quantified and analyzed based on their protein concentration. Therefore, normalizing BMV quantity by particle number in functional assays enables comparisons to be made between BMVs from different bacterial strains, species, stages, or conditions of bacterial growth and between biological studies, facilitating a deeper understanding of differences in the biological functions of BMVs based on their cargo content.

## DISCUSSION

The multifaceted functions of BMVs are attributed to the diverse cargo they contain (50). Therefore, examining how BMV composition influences their biological activities is critical to understanding their functions. The findings of this study highlight the complexities of BMV quantification. We show that protein assays vary in their quantification of BMVs depending on the type of protein assay used (Fig. 1), that BMV quantification by protein assay can reduce the ability to differentiate differences in BMV cargo content, including protein, DNA, and RNA, compared to BMV quantification by particle number (Fig. 2 and 4), and that this can significantly alter the outcome of downstream immunological assays (Fig. 5 and 6). Furthermore, our findings identifying that BMV cargo content varies significantly between bacterial species and stages of bacterial growth raises important considerations for the analysis of BMV functions, in particular comparing the functions of BMVs between biological studies that have different BMV production, isolation, and quantification methods, as these parameters influence BMV cargo content.

Our data reveal that there are variations in the quantification of BMVs using Coomassie-, copper-, or fluorescence-based protein assays, which affect BMV quantification and result in variability of subsequent immunological assays (Fig. 1). Furthermore, it appears that there is also variability in the quantification of BMVs produced by different bacteria when using each of the protein assays (Fig. 1). Although these findings suggest that a single protein assay cannot be selected as the ideal assay for the quantification of BMV-associated proteins from all bacterial species, they indicate that copper- and fluorescence-based protein assays are more sensitive than Bradford assay for the quantification of BMV proteins (Fig. 1). Consistent with our findings, previous studies have also shown that protein quantification of various biological samples can vary significantly among protein assays, depending on the size, composition, and covalent modifications of the proteins within the sample (51, 52). BMVs contain a multitude of proteins, all of different size, sequence, and covalent modifications (3, 53). These factors may influence the ability of different protein assays to quantify BMV proteins and may be a possible reason for the variability in the quantification of their protein cargo.

In this study, we used NTA to quantify BMVs based on particle number, since NTA is not dependent on cargo content that varies between bacterial species, strains, and growth conditions. By performing epithelial cell stimulation assays based on BMV number rather than BMV protein amount, we demonstrated that subtle differences in BMV cargo contents can have significant effects on their immunogenic functions (Fig. 5 and 6). However, as with many assays, NTA has potential limitations in measuring BMV number, as NTA measures particles within a solution and therefore not BMVs *per se*. Furthermore, particles contained within bacterial growth media can also be detected by NTA, highlighting the importance of BMV purification and verification of BMV purity by TEM to maximize the accuracy of BMV quantification by NTA. In addition, NTA can be used to improve BMV detection within a sample where purity is not possible, such as in biological samples, and to distinguish them from other particles within the sample by the use of BMV-specific antibodies, if the species producing the BMVs is known (54). Therefore, identifying BMV-specific markers, such as those specific to Escherichia coli BMVs (46), will further aid in the development of highly specific methods for the quantification of BMVs within such samples.

In addition to NTA, particle number can also be measured using several other techniques not examined in this study, including high-resolution nano-flow cytometry (17), resistive pulse sensing (RPS) (55), cryo-electron microscopy (56), and surface plasmon resonance combined with atomic force microscopy (57). Each of these techniques has advantages and limitations and can vary in accuracy depending on particle size range and concentration (37). Thus, different BMV quantification methods may be more suitable for different BMV studies, depending on the amount, purity, or size of BMVs in a given preparation. For example, one study reported the use of high-resolution nano-flow cytometry to quantify BMVs and subsequently compared the responses of host cells stimulated with BMVs based on particle number or protein assay (17). In agreement with our findings, the authors showed that different responses of pattern recognition receptors to BMVs from each pathogen were observed when stimulations were performed based on particle number but not protein assay (17).

Furthermore, we have previously shown that BMV size can also influence their cargo content and therefore their biological functions (29). This suggests that particle number alone may not always reflect the functional differences between BMVs from different preparations but that BMV size range should also be considered. Separation of BMVs based on size and subsequent quantification and size enumeration by NTA (29) can therefore give insight into the functional differences between BMV populations of different sizes. In addition to size, BMV preparations can also contain different types of BMVs, such as outer-inner membrane vesicles (O-IMVs) (58) or BMVs produced by explosive cell lysis (59), which have been shown to differ in DNA content compared to BMVs produced by blebbing from the outer membrane (58, 59). Therefore, in addition to particle enumeration, examination of BMV morphology by TEM and cargo examination will provide valuable insight into BMV functions. Taken together, these findings highlight that examination of particle number, size range, morphology, and cargo content of BMVs can broaden our understanding of BMV functionality, improve experimental reproducibility, and enable biological comparisons across different biological studies within the BMV field.

In the interest of ensuring accurate characterizations of eukaryotic EVs, a set of standard guidelines have been developed detailing the requirements for the production, quantification, and examination of EVs, termed the minimal information for studies of extracellular vesicles (MISEV) guidelines (37). Supporting our findings, the MISEV guidelines indicate that the quantification of EVs based on their cargo alone does not correlate with EV numbers (37). Therefore, the MISEV guidelines encourage a multifaceted approach to EV quantification, which includes reporting the ratios of cellular components to particles, such as proteins to particles or lipids to particles, in addition to the quantification estimates of EVs as a measure of EV purity and quantity (37). This suggests that an integrative approach can also be developed for the quantification of BMVs that may promote the development of an equivalent standard guidelines for the production, quantification, and examination of BMVs to ensure rigor, clarity, and reproducibility in the rapidly growing BMV field. The findings of this study therefore provide insights into the complexities of BMV quantification that should be considered for the development of such standard guidelines and by researchers conducting BMV-based studies.

The experimental rigor and clarity that would ensue from the development of standard guidelines for BMV production, quantification, and examination would provide multiple benefits to the BMV field. In addition to deepening our understanding of the correlation between BMV content and functions, it would also propel the development of BMVs as therapeutic platforms or novel vaccines. Studies examining different production methods of the current licensed BMV-based vaccine against Neisseria meningitidis serogroup B have shown that bacterial growth stage, pH of the culture media, and method of BMV isolation significantly affected the protein content of BMV vaccines (47, 60). Furthermore, bacterial growth stage and the pH of culture media have been shown to influence the degree of bacterial lysis during BMV production (60), which can affect the mechanism of BMV biogenesis and therefore BMV content (59). An integrative approach to BMV quantification may, therefore, advance the pursuit of novel BMV-based therapeutics by helping to identify specific bacterial strains, growth conditions, growth stages, or methods of BMV isolation that yield greater amounts of desired cargo, such as immunogenic antigens for vaccine development (61), regulatory RNA for anti-cancer therapies (62), or antimicrobial compounds for the development of nanoantibiotics (63). In addition, this can reduce the issue of batch-to-batch variation between BMV preparations, which remains a hurdle for BMV-based therapies (64, 65).

Collectively, this study reveals the complexities of BMV quantification and the impact that BMV quantification based on protein content using various protein assays can have on the outcomes of immunological and biological studies. Furthermore, these findings identify the complexity of BMV composition based on their strain of origin and the growth stage from which they were isolated. Finally, this work also highlights the need for a standardized, integrative approach to BMV quantification that takes into consideration several variables that influence BMV cargo content and therefore their function, including bacteria of origin, bacterial stage and conditions of growth, sample purity, particle number, BMV size, and cargo content. These insights provide considerations for the development of such standard guidelines, whose implementation will advance our understanding of the multifaceted functions of BMVs that are attributed by their various cargo, and ultimately bring consistency and uniformity to the rapidly growing field of BMV research.

## MATERIALS AND METHODS

### Bacterial strains and culture conditions.

Helicobacter pylori strains 251 Δ*cag*PAI and 26695 were routinely cultured on horse blood agar (HBA) supplemented with 0.2% Skirrow’s selective supplement at 37°C in microaerophilic conditions (CampyGen, Oxoid) (5, 66). Staphylococcus aureus NCTC 6571 was maintained at 37°C on HBA (7), and Pseudomonas aeruginosa PAO9505 was maintained at 37°C on nutrient agar as described previously (67).

### BMV isolation and purification.

H. pylori cultures for the purpose of BMV isolation were cultured in brain heart infusion (BHI) broth supplemented with 0.6% (wt/vol) β-cyclodextrin (ThermoFisher Scientific) at a starting optical density at 600 nm (OD_600_) of 0.1 and grown at 37°C shaking at 120 rpm in microaerophilic conditions (CampyGen, Oxoid) for 16 h to early-exponential stage, as described previously (29). To obtain BMVs from late-exponential or stationary stage of bacterial growth, H. pylori cultures were inoculated at a starting OD_600_ of 0.1 and incubated for 48 h or 72 h, as described previously (4). S. aureus was inoculated at a starting OD_600_ of 0.1 and grown in BHI for 16 h at 37°C shaking at 180 rpm (7), and P. aeruginosa was inoculated at a starting OD_600_ of 0.1 and grown in nutrient broth (Oxoid) for 16 h at 37°C and 180 rpm. BMVs were isolated from all cultures as described previously (4, 5, 29). Briefly, bacteria were pelleted at 4,000 × *g* for 30 min at 4°C (Heraeus MegaFuge 4R, ThermoFisher Scientific) and the supernatants were filtered through 0.22-μm-pore-size filters (Millipore). BMVs were pelleted by ultracentrifugation at 100,000 × *g* for 2 h at 4°C (Hitachi CP100NX). The resulting BMV pellet was purified by OptiPrep (iodixanol; Sigma-Aldrich) density gradient separation (4, 7). Fractions containing BMVs were identified by electron microscopy, pooled, and washed twice with phosphate-buffered saline (PBS) by ultracentrifugation at 100,000 × *g* for 2 h. The resulting purified BMV pellet was resuspended in PBS and stored at −80°C until required.

### Quantification of BMVs using protein assays.

BMVs were quantified based on their protein content using the colorimetric assays Bradford (Bio-Rad Laboratories), Lowry DC (Bio-Rad Laboratories), and BCA (Pierce) or the fluorometric Qubit protein assay (ThermoFisher) as indicated, according to the manufacturer’s guidelines. For colorimetric protein assays, protein standards were prepared using bovine serum albumin (BSA) diluted in PBS, and absorbance was measured at 595 nm (Bradford), 650 nm (Lowry), or 652 nm (BCA) using a FLUOstar OPTIMA plate reader (BMG Labtech). For enumeration of protein using Qubit, fluorescence was measured using a Qubit 3.0 fluorometer that had been calibrated using Qubit protein standards (ThermoFisher).

### BMV quantification using nanoparticle tracking analysis.

Direct quantification of BMVs was performed by NTA using a NanoSight NTA 3.2 and NanoSight NS300 NTA software version 3.2 equipped with a 430 nm laser (Malvern Instruments), as described previously (4, 7). Samples were diluted in Dulbecco’s PBS (DPBS) to give an average of 20 to 100 particles per field, and the instrument was flushed with DPBS and air between samples. NTA of all BMV samples was performed in triplicate by capturing 60-s reads with a flow rate of 50 at 25°C. Capture settings were set to camera gain of 1, camera level 16, slider shutter 1,300, and slider gain 512. Analysis settings were set to a detection threshold of 5, with minimum particle size, blur, and minimum track length set automatically. Data outputs were generated using NanoSight NS300 NTA software version 3.2 (Malvern Instruments). The average number of particles at each binned center in the experiment summary output was adjusted by the dilution factor. The mean of 3 biological replicates was plotted as particle size versus number of particles per milliliter ± standard error of the mean (SEM) using GraphPad Prism 8 Software (GraphPad Software).

### Transmission electron microscopy.

Sample preparation for TEM was performed as described previously (11). Briefly, BMVs were coated onto carbon-coated 400 mesh copper grids (ProSciTech), fixed in 1% (wt/vol) glutaraldehyde (Sigma) in PBS, stained with 2% (wt/vol) uranyl acetate (ProSciTech) (pH 7.0), and coated with 2% (wt/vol) methyl-cellulose (Sigma) in 0.4% (wt/vol) uranyl acetate (pH 4.0). Samples were viewed using a JEOL JEM-2100 transmission electron microscope (JEOL, Japan) operated at 200 kV fitted with a Valeta 4 MP CCD camera (Emsis, Germany).

### Comparison of the species, strain, and growth stage differences in BMV protein, DNA, and RNA cargo content.

The protein, DNA, and RNA content of BMVs were quantified using the Qubit protein assay, Qubit high sensitivity DNA assay, or Qubit high sensitivity RNA assay kits, respectively (ThermoFisher). Protein, DNA, and RNA were measured using a Qubit 3.0 fluorometer. Protein, DNA, and RNA concentrations were normalized to 1 × 10^10^ BMVs for all bacterial strains and growth stages from which BMVs were isolated.

### Cell culture stimulations.

Human embryonic kidney (HEK293; ATCC CRL-1573) cells were cultured and transfected using established techniques (5, 7). HEK293 cells were seeded at 2 × 10^5^ cells/mL and transfected with reporter constructs IgK luciferase (5) and dTK renilla (Promega). Transfected cells were stimulated for 8 h with BMVs and lysed with reporter lysis buffer (Promega), and luminescence was measured using a FLUOstar OPTIMA plate reader (BMG Labtech).

Gastric adenocarcinoma cells (AGS; ATCC CRL-1739) were cultured as described previously (5, 29). AGS cells were seeded at 2 × 10^5^ cells/mL in 24-well plates (Greiner) and stimulated with BMVs for 24 h in serum-free RPMI (6). Human interleukin-8 (IL-8) in cell culture supernatants was detected by enzyme-linked immunosorbent assay (IL-8 ELISA, BD Biosciences) and measured using a FLUOstar OPTIMA plate reader (BMG Labtech).

Commercially available HEK-Blue cell lines Null, hTLR7, and hTLR9 containing an inducible NF-κB/AP1 secreted alkaline phosphatase (SEAP) reporter (InvivoGen) were maintained as described previously (7). HEK-Blue cells were seeded at 2 × 10^5^ cells in 200 μL in 96-well plates (Griener) and stimulated for 24 h with an increasing number of purified early-exponential BMVs. Positive controls were 1 pg/mL R848 (resiquimod; InvivoGen) for TLR7 cells and 5 nM CpG ODN (InvivoGen) for TLR9 cells. Cell culture supernatants were assayed for secreted alkaline phosphatase (SEAP) activity by incubating 20 μL of supernatant with 180 μL of QUANTI-Blue solution (InvivoGen) at 37°C, and SEAP activity was measured at 620 nm using a FLUOstar OPTIMA reader (BMG Labtech).

### Statistical analyses.

Data analysis was performed using GraphPad PRISM 8. All data are presented as mean ± standard error of the mean (SEM) of three biological replicates unless otherwise stated. Statistical analyses were performed using data from three or more biological replicates, using the one-way analysis of variance (ANOVA) with Tukey’s multiple-comparison test or *t* test as indicated.
